# Recruiting Hard-to-Reach Populations for Survey Research: Using Facebook and Instagram Advertisements and In-Person Intercept in LGBT Bars and Nightclubs to Recruit LGBT Young Adults

**DOI:** 10.2196/jmir.9461

**Published:** 2018-06-18

**Authors:** Jamie Guillory, Kristine F Wiant, Matthew Farrelly, Leah Fiacco, Ishrat Alam, Leah Hoffman, Erik Crankshaw, Janine Delahanty, Tesfa N Alexander

**Affiliations:** ^1^ RTI International Research Triangle Park, NC United States; ^2^ US Food and Drug Administration Center for Tobacco Products Silver Spring, MD United States; ^3^ Battelle Memorial Institute Aberdeen, MD United States

**Keywords:** sexual minorities, social media, public health, tobacco, survey methods, transgender persons

## Abstract

**Background:**

Tobacco public education campaigns focus increasingly on hard-to-reach populations at higher risk for smoking, prompting campaign creators and evaluators to develop strategies to reach hard-to-reach populations in virtual and physical spaces where they spend time.

**Objective:**

The aim of this study was to describe two novel recruitment strategies (in-person intercept interviews in lesbian, gay, bisexual, and transgender [LGBT] social venues and targeted social media ads) and compares characteristics of participants recruited via these strategies for the US Food and Drug Administration’s *This Free Life* campaign evaluation targeting LGBT young adults who smoke cigarettes occasionally.

**Methods:**

We recruited LGBT adults aged 18-24 years in the United States via Facebook and Instagram ads (N=1709, mean age 20.94, SD 1.94) or intercept in LGBT social venues (N=2348, mean age 21.98, SD 1.69) for the baseline evaluation survey. Covariates related to recruitment strategy were age; race or ethnicity; LGBT identity; education; pride event attendance; and alcohol, cigarette, and social media use.

**Results:**

Lesbian or gay women (adjusted odds ratio, AOR 1.88, 95% CI 1.54-2.29, *P*<.001), bisexual men and women (AOR 1.46, 95% CI 1.17-1.82, *P*=.001), gender minorities (AOR 1.68, 95% CI 1.26-2.25, *P*<.001), and other sexual minorities (AOR 2.48, 95% CI 1.62-3.80, *P*<.001) were more likely than gay men to be recruited via social media (than intercept). Hispanic (AOR 0.73, 95% CI 0.61-0.89, *P*=.001) and other or multiracial, non-Hispanic participants (AOR 0.70, 95% CI 0.54-0.90, *P*=.006) were less likely than white, non-Hispanic participants to be recruited via social media. As age increased, odds of recruitment via social media decreased (AOR 0.76, 95% CI 0.72-0.80, *P*<.001). Participants with some college education (AOR 1.27, 95% CI 1.03-1.56, *P*=.03) were more likely than those with a college degree to be recruited via social media. Participants reporting past 30-day alcohol use were less likely to be recruited via social media (AOR 0.33, 95% CI 0.24-0.44, *P*<.001). Participants who reported past-year pride event attendance were more likely to be recruited via social media (AOR 1.31, 95% CI 1.06-1.64, *P*=.02), as well as those who used Facebook at least once daily (AOR 1.43, 95% CI 1.14-1.80, *P*=.002). Participants who reported using Instagram at least once daily were less likely to be recruited via social media (AOR 0.73, 95% CI 0.62-0.86, *P*<.001). Social media recruitment was faster (incidence rate ratio, IRR=3.31, 95% CI 3.11-3.52, *P*<.001) and less expensive (2.2% of combined social media and intercept recruitment cost) but had greater data quality issues—a larger percentage of social media respondents were lost because of duplicate and low-quality responses (374/4446, 8.41%) compared with intercept respondents lost to interviewer misrepresentation (15/4446, 0.34%; *P*<.001).

**Conclusions:**

Social media combined with intercept provided access to important LGBT subpopulations (eg, gender and other sexual minorities) and a more diverse sample. Social media methods have more data quality issues but are faster and less expensive than intercept. Recruiting hard-to-reach populations via audience-tailored strategies enabled recruitment of one of the largest LGBT young adult samples, suggesting these methods’ promise for accessing hard-to-reach populations.

## Introduction

### Background

Public education campaigns aimed at educating the public on the risks of tobacco use are increasingly targeting specific segments of the population who are at risk for tobacco use and hard to reach via traditional methods [1,2]. In the United States, the Center for Tobacco Products (CTP) at the US Food and Drug Administration (FDA) implements a number of these campaigns as part of its mission to educate the public on the harms of tobacco use. Each campaign addresses at-risk, hard-to-reach populations (ie, populations that are difficult to reach for inclusion in surveys via traditional survey research recruitment methods) and delivers compelling content relevant to that specific target population. These campaigns include FDA’s flagship campaign, *The Real Cost*, that targets young people aged 12-17 years who are at risk for initiating cigarette smoking or are experimenting with smoking (ie, have smoked fewer than 100 cigarettes in their lifetime) and includes a component that focuses on educating hard-to-reach rural male youth at risk for using smokeless tobacco [1]. FDA’s *Fresh Empire* campaign targets African American, Hispanic, Asian or Pacific Islander, and multiracial youth who are influenced by the hip hop peer crowd and at risk for cigarette smoking [2]. *This Free Life* is FDA’s public education campaign focusing on lesbian, gay, bisexual, and transgender (LGBT) young adults aged 18 to 24 years who smoke cigarettes occasionally [3].

LGBT young adults are hard to reach [4] and have significant tobacco use disparities compared with non-LGBT young adults [5-7], being almost twice as likely to use tobacco as their non-LGBT peers [8,9]. Elevated risk of tobacco use among LGBT individuals has been attributed to LGBT-targeted tobacco product marketing [5,7,10-12] and minority stress (ie, strain resulting from stigma and discrimination associated with having a minority identity) [13-17]. LGBT minority stress increases risk of depression, alcohol and other substance abuse, homelessness, and poorer health, which are all factors associated with tobacco use [6,15,16,18-22].

### Survey Recruitment Methods for Hard-To-Reach Populations

Developing tobacco public education campaigns for hard-to-reach populations such as LGBT young adults also comes with the challenge of reaching these populations to evaluate whether the campaign is effectively educating them about the harms of tobacco use. Researchers are increasingly turning to innovative strategies for recruiting hard-to-reach populations vs traditional methods [23-34]. One strategy that researchers use to recruit young adults involves conducting intercept interviews in social venues (eg, bars and nightclubs) where the target population spends time. A number of researchers have used this strategy to recruit young adults who are at a higher risk for smoking and alcohol use [24-28]. Furthermore, Fallin and colleagues used this strategy to successfully recruit LGBT young adults in bars and nightclubs [23].

A second strategy that has become popular for recruiting hard-to-reach populations for survey research is the use of targeted ads on social media platforms such as Facebook, Instagram, and Twitter [29,31-37]. Social media platforms possess massive quantities of user data that allow for highly specific targeting of ads to hard-to-reach populations on multiple features such as age, gender, location, interests, and relationship preferences (women interested in women, women interested in women and men, men interested in men, and men interested in women and men). A growing number of studies have successfully recruited hard-to-reach populations via social media, including young adult and adolescent smokers in the United States [29,31-32], adult electronic cigarette users [35], adult gay men [33,38,39], gay and bisexual youth [30], adolescent and young adult women in Australia [37,40], and HIV-positive adults in the United States [34].

### This Study

In the present research, we focus on data collected for the evaluation of FDA’s *This Free Life* tobacco public education campaign targeting LGBT young adults. *This Free Life* engages with the target group in 12 designated market areas (DMAs) in the United States through multiple channels including social media and LGBT-specific digital sites, streaming radio, LGBT print media, branded promotions at LGBT events and social venues, and out-of-home media such as signage at bus stops in areas where LGBT young adults are likely to socialize. From a campaign evaluation perspective, we consider these 12 DMAs to be treatment DMAs and compare them against data we collected for the evaluation in 12 control, or comparison, DMAs where no events occur, and minimal campaign advertising occurs. The data we present in this paper come from the baseline wave of data collection that occurred immediately before the launch of the *This Free Life* media campaign in the 12 treatment DMAs in the United States. We employed two unique strategies to recruit LGBT young adults for this study. First, we conducted in-person intercept screening interviews using tablet devices in social venues such as bars and nightclubs that we identified as popular among LGBT young adults in each of the 24 DMAs. Second, we used social media ads on Facebook and Instagram that linked to a Web screening instrument. For social media ads, we used targeting tools and targeted ad content to recruit LGBT young adults in the 24 DMAs. In this study, we compare the cost and time efficiency of recruitment and quality of data gleaned between the two novel methods. Considering that findings from previous research indicate that data collection via social recruitment is more time-efficient than traditional methods (eg, email invitations and print ads) [36], in combination with the fact that a large amount of resources and time are required for intercept recruitment, we hypothesize that the sample will be recruited more quickly and at a lower cost via social media than intercept. Furthermore, we explore how the LGBT young adults we recruited via these methods differ by LGBT identity, demographics, cigarette use, alcohol use, social media use, and participation in LGBT culture.

## Methods

### Participants

Eligible participants were young adults, aged 18 to 24 years, who self-identified as LGBT, and lived in one of the 24 DMAs in the United States (N=4057, mean age 21.54, SD 1.87). We recruited participants from February 2016 to May 2016 before the launch of FDA’s *This Free Life* media campaign. The study was approved by RTI International’s institutional review board.

### Recruitment Method

We recruited participants using either in-person intercept interviews in LGBT social venues (N=2348) or via social media ads (N=1709).

#### Intercept Recruitment

We intercepted and asked participants to complete a screener (ie, screening instrument) in LGBT social venues that we identified through Web searches and recommendations from local field staff. We discuss full details of intercept recruitment in the Procedure section.

#### Social Media Recruitment

We used Facebook and Instagram ads for social media recruitment. Facebook and Instagram ads run through the same platform and ad sets; thus, content and other targeting features are identical. To recruit a broad sample that represented a range of LGBT subgroups, we used three ad sets with different images and targeting criteria. The first was a male-centric ad set targeted to men whose relationship interest was in men or men and women. The second was a female-centric ad set targeted to women whose relationship interest was in women or women and men. The third ad set targeted gender minorities (eg, transgender and genderqueer) and used keywords representing transgender and other gender minority celebrities, historic figures, causes (eg, Transgender Student Rights), and outreach groups. Several of the study’s authors identified keywords for the gender minority ad set through formative research conducted during the development stages of the media campaign content with focus groups of LGBT young adults. We outline targeting strategies unique to each of the ad sets in Table 1. All ad sets targeted English-speaking young adults, aged 18 to 24 years, and who live in one of the 24 evaluation DMAs.

**Figure 1:**
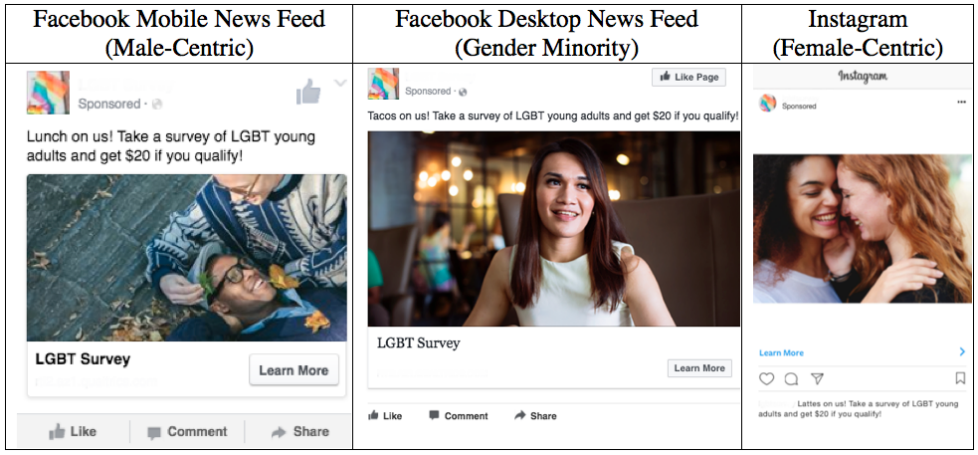
Facebook and Instagram advertisement examples.

Facebook and Instagram ads consisted of a brief text description of the survey and incentive amount for qualifying participants (eg, “Breakfast on us! Take a survey of LGBT young adults and get $20 if you qualify!”), a reference to the Facebook page or Instagram account associated with the ads, and an image. Figure 1 shows sample Facebook and Instagram ads for male-centric, gender minority, and female-centric campaigns. We crafted text descriptions for all ad sets to appeal to the target audience of young adults, referencing goods and services that young adults would likely want (and realistically be able) to purchase with the US $20 gift card that they would receive if they qualified and completed the survey (eg, “Tacos on us! …”; “Cupcakes on us! …”; “Treat yo self! …”; “Burgers on us! …”; and “Lattes on us! …”). We chose images to represent the specific target audience for each of the three ad sets, with the male-centric ads including images of male young adult couples and an LGBT pride flag, female-centric ads including images of female young adult couples and an LGBT pride flag, and gender minority ads including images of transgender young adults. We also created an Instagram account that was associated with Instagram recruitment ads. Images from all ad sets were included in the Facebook page and Instagram account for the study.

**Figure 2:**
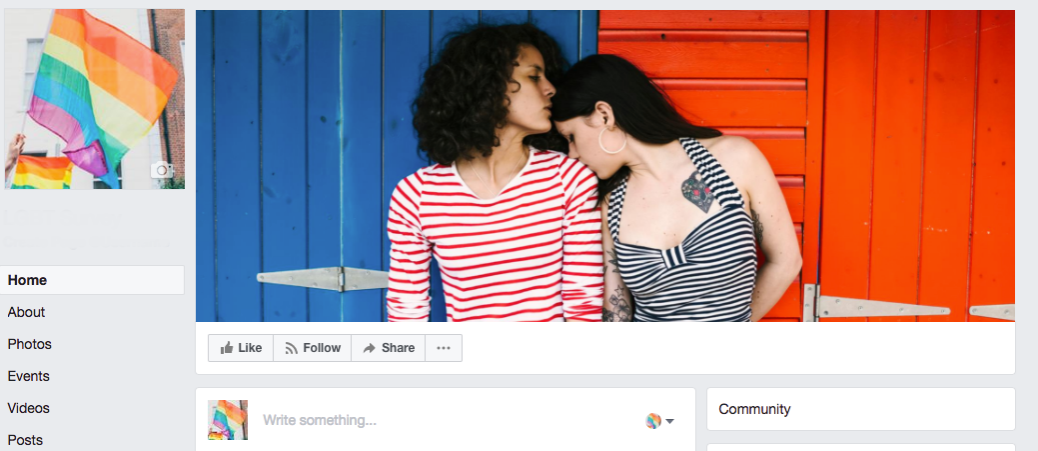
Facebook page. Facebook page name has been removed to avoid compromising ongoing waves of data collection for the campaign evaluation.

To distribute Facebook ads, Facebook requires that a Facebook page be associated with ads (see Figure 2). We created a Facebook page to use for the study and associated it with Facebook recruitment ads.

**Table 1:** Facebook and Instagram targeting criteria.

Facebook or Instagram targeting strategy	Targeting criteria
Male-centric	Interested in Men Men and women
Female-centric	Interested in Women Women and men
Gender minority	Keywords: Against Me!, Caitlyn Jenner, Chaz Bono, Fallon Fox, Janet Mock, Jenna Talackova, Laura Jane Grace, Laverne Cox, Lea T, Lili Elbe, National Center for Transgender Equality, transgender, Transgender Law Center, Transgender Student Rights, Transgender youth, Wendy Carlos

Ads were displayed on desktop and laptop computers (as sidebar or Facebook Desktop News Feed ads), smartphones (as mobile Facebook News Feed ads), on Instagram (via the mobile Instagram app), and third-party apps in which Facebook places ads.

### Dependent Variable

The dependent variable in this study was recruitment method (in-person intercept vs social media) for number of completed surveys.

### Procedure

#### Intercept Recruitment

We fielded the baseline intercept recruitment from February 2016 to May 2016. Trained field staff visited the potential venues to confirm the venue’s popularity with the target audience and, as appropriate, gain permission to conduct the intercept study there. Field supervisors followed up with recruited venues via email to confirm arrangements and schedules. Within each DMA, two to five local data collectors attended a 5-hour in-person training before collecting data. Once trained, groups of two to five interviewers visited the recruited venues at the agreed-upon date and time. We designated one of the interviewers as the recruiter. Interviewers intercepted potential participants at venues where the recruiter approached patrons who appeared to be in the target age range and used a standardized script to introduce themselves, describe the screening process and incentive amount, prescreen patrons for age eligibility (ie, verify that participants were younger than 25 years), and ask patrons who stated they were in the age range of 18 to 24 years to complete the 5-min, self-administered screener on a tablet. To promote data quality, the trainer accompanied the data collectors on the first 2 to 5 nights of data collection to monitor compliance with data collection protocols and provide additional on-the-job training when needed. We also used global positioning system (GPS) coordinates and timing data captured within the screeners to identify screeners of questionable authenticity (ie, screeners taken at times and in locations that did not align with the times and locations of data collection, suggesting interviewer misrepresentation). The recruiter was responsible for maintaining tallies of the outcome of this contact (ie, refused, not aged 18-24 years, and agreed to participate).

As patrons agreed, a data collector helped the patron launch the screener to determine eligibility for participating in the main survey that included questions about age, home zip code, LGBT identity, and cigarette use. Data collectors provided each intercept respondent US $10 in cash for completing the screener and provided respondents who screened as eligible with a study information card with details about next steps. For those who screened as eligible, within 2 days an invitation was sent via SMS text message (short message service, SMS) or email (based on the participant’s stated preference) to complete the full 30-min Web survey. Participants who clicked on the Web link for this survey were first directed to a brief consent form. Those who consented completed the main survey that included questions about demographics; tobacco, alcohol, and social media use; participation in and identification with LGBT culture; and tobacco-related knowledge, attitudes, and beliefs. Those who completed the survey received a US $20 digital gift card with a US $5 bonus (total of US $25) for completing the full survey within 2 days of receiving the first invitation. Respondents who did not respond to the first invitation received up to three additional prompts, spaced every other day.

#### Social Media Recruitment

We conducted the male- and female-targeted social media recruitment during 1 week in March 2016 and the gender minority recruitment over 5 days from April 2016 to May 2016. We made first contact with potential participants via Facebook and Instagram ads. Participants clicked on ads that sent them to a link for the same screening instrument that intercept respondents completed. Social media participants did not receive an incentive for completing the screener. Eligible participants, identified via responses to the screener, proceeded directly to the same consent form provided to intercept participants. Consenting participants completed the same survey as intercept participants. Participants recruited via social media received a US $20 digital gift card for their participation. We did not provide the US $5 bonus to social media respondents because eligible participants proceeded directly to the main survey from the screener.

### Predictor Variables

Independent variables were age; LGBT identity—lesbian (ie, cisgender lesbian or gay women), gay (ie, cisgender gay men), bisexual (ie, cisgender bisexual men and women), gender minority (ie, transgender, genderqueer, and gender-variant men and women), other sexual minority (eg, pansexual, omnisexual, and trisexual)—education; race or ethnicity; past 30-day cigarette use; past 30-day alcohol use; past-year pride event attendance; and social media use (ie, Facebook and Instagram use frequency). Only participants who are not gender minorities or other sexual minorities were grouped by their sexual identity.

### Statistical Analysis

First, we compared the two recruitment strategies on the total cost and time efficiency of data collection. Data on cost of recruitment were available only as a proportion of total recruitment costs for each recruitment method; thus, our cost comparisons are limited to simple descriptive comparisons of the proportion of total cost of recruitment between intercept and social media.

To ascertain which of the two recruitment methods was more time-efficient in recruiting participants, we conducted a Poisson regression on the count of people who completed the full survey, with weeks required to recruit the sample of completed surveys for each recruitment method included as an offset variable and method of recruitment as the predictor variable [35,36]. The inclusion of weeks to survey completion as an offset variable allowed us to calculate recruitment efficiency as an incidence rate ratio (IRR) for weeks required for each recruitment method to recruit a single participant.

We then compared intercept and social media data collection on the quality of data collected and their effectiveness in identifying eligible participants. We cleaned the raw dataset to remove low-quality (ie, non-US-based internet protocol (IP) addresses and IP addresses known to be associated with malicious software or services) and duplicate responses from the social media data (ie, multiple responses from the same IP address in a small window of time and responses associated with an email address that is at least 80% similar to an email address already associated with a completed Web survey) and interviewer misrepresentation from the intercept data (ie, generation of fake responses as detected by GPS coordinates and timing data associated with completed screeners). We used *t* tests to compare the percentage of data lost because of low-quality or duplicate responses from social media with the percentage of data lost because of interviewer misrepresentation from intercept. We used a series of unpaired sample means tests to compare the percentage of people from social media vs intercept who completed screeners, were eligible, and completed the baseline survey.

Third, we compared the characteristics of participants (see the predictor variables described previously) recruited via the two methods. We used descriptive statistics (means, frequencies, and percentages) to describe the sample characteristics for each recruitment method. We then conducted bivariate analyses to determine differences between participants recruited via intercept vs social media for each of the predictor variables. We created the final multivariate model using predictor variables that we found to be related to recruitment method in bivariate analyses (at the *P*<.25 level following methods from Hosmer and Lemeshow [41]). The final model was a multivariate logistic regression with recruitment method as the outcome variable and the following predictor variables: age, education, race or ethnicity, LGBT identity, past 30-day cigarette and alcohol use, past-year pride event attendance, and Facebook and Instagram use frequency. Analyses were run in Stata 14 (StataCorp, LLC).

## Results

### Intercept Recruitment Screener Completion Rate

For intercept recruitment, we approached 9552 individuals who appeared to be within the eligible age range in venues and asked them to complete the screener. Of those asked, 7375 completed the screener, resulting in a 77.21% screener completion rate among those approached.

### Social Media Recruitment Advertisement Performance and Screener Completion Rate

The Facebook and Instagram ads used to recruit participants reached a total of 324,959 individual users (exposed to ads at least once): 81,312 with female-centric ads, 44,802 with male-centric ads, and 204,614 with gender minority ads. Ads resulted in 7249 total clicks, with 2225 clicks on female-centric ads (2225/81,312, 2.74% of people exposed to ads, clicked on links), 1558 clicks on male-centric ads (1558/44,802, 3.48% of people exposed to ads, clicked on links), and 3466 clicks on gender minority ads (3466/204,614, 1.69% of people exposed to ads, clicked on links). It is important to note that because gender minority participants are a particularly hard-to-reach subpopulation within the LGBT young adult population, we devoted a larger budget and more run time to gender minority ads to generate a more diverse sample.

Of social media respondents who clicked on links, 6611 completed the screener, resulting in a 91.20% (6611/7249) screener completion rate for people who clicked on ads. Due to privacy features on the ad platforms, we cannot tie link clicks from specific ad sets to completed screeners; thus, the screener completion rate could only be generated for ad sets in combination.

### Cost and Time Efficiency Comparisons Between Intercept and Social Media Recruitment

Descriptive comparisons of recruitment costs between recruitment methods show that social media recruitment is less expensive than intercept recruitment, with social recruitment making up just 2.2% of total recruitment costs and intercept recruitment making up 97.8% of total recruitment costs. This substantial difference is largely because of the large number of resources required to conduct intercept recruitment, as costs include labor (for staff training and time in the field recruiting participants), mileage for traveling to and from recruitment venues, screener incentives, and miscellaneous expenses (ie, parking and cover costs for venue entry), whereas the only cost for social media recruitment is the cost of placing ads on Facebook and Instagram.

**Figure 3:**
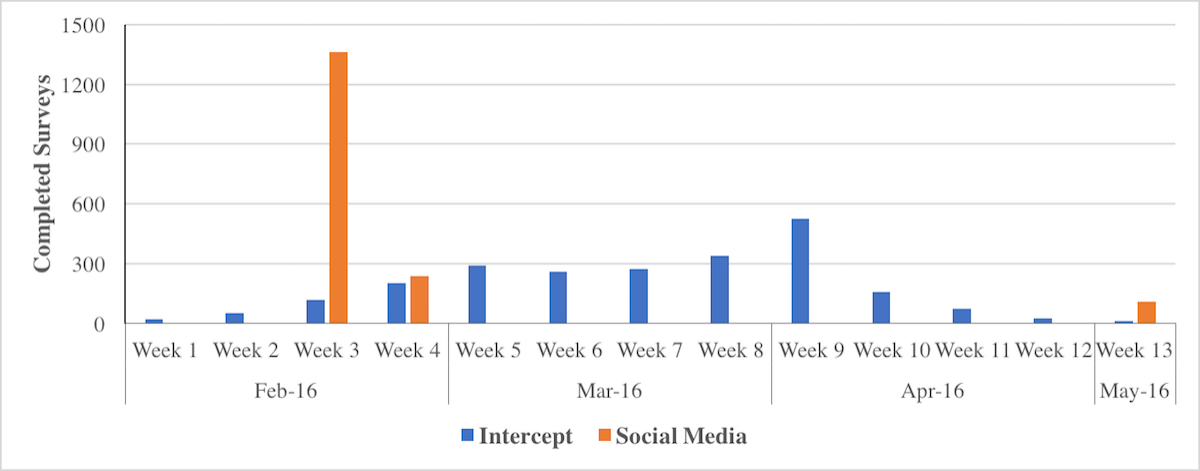
Number of surveys completed by week for intercept and social media recruitment.

Social media recruitment is also more time-efficient than intercept recruitment, as we found that the IRR for time to survey completion was 3.31 (95% CI 3.11-3.52) times faster for social media participants than intercept participants for the full data collection period (*P*<.001). Figure 3 illustrates the number of surveys completed for each recruitment method by week of data collection. Time required for intercept recruitment includes training, travel time to and from recruitment venues, and time spent recruiting participants in venues, whereas social media recruitment time includes the total number of weeks that ads were run to recruit participants. As is the case with cost, these large differences in time efficiency are attributed to additional time required for intercept data collection.

### Data Quality Comparisons Between Intercept and Social Media Recruitment

Social media recruitment was more vulnerable to data quality issues than intercept. During data cleaning, we dropped a significantly larger percentage of social media respondents because of low-quality and duplicate responses (374/4446, 8.41%) than intercept respondents dropped because of interviewer misrepresentation (15/4446, 0.34%; *P*<.001). Denominators represent the sample N before we dropped cases from the analytic sample (denominator = analytic N + N dropped for low-quality or duplicate responses + N dropped for interviewer misrepresentation).

### Completed Screeners, Eligibility, and Survey Completion

Across the full sample, more than half of the people who completed screeners were eligible to participate in the main survey (7965/13986, 56.95%), and half of eligible participants (ie, young adults aged 18-24 years who self-identified as LGBT and reported living in a zip code in one of the 24 study DMAs) completed the main survey (4057/7965, 50.93%). A significantly larger percentage of participants who completed the screener via intercept (4608/7375, 62.48%) were eligible to complete the main survey than participants recruited via social media (3357/6611, 50.78%; *P*<.001). The proportion of eligible participants who completed the survey did not differ between recruitment methods. Results are presented in Table 2.

### Sample Characteristics

We describe the sample in Table 3. We recruited more than half of the sample via intercept (2348/4057, 57.88%). The mean age of the sample was 22 years (SD 1.87), with participants recruited via intercept being significantly older than those recruited via social media (*P*<.001). Participants who self-identified as gay men made up the largest proportion of the overall sample (1822/4057, 44.91%), followed by lesbian or gay women (882/4057, 21.74%), bisexual men (219/4057, 5.40%) and women (639/4057, 15.75%), gender minorities (342/4057, 8.43%), and other sexual minorities (152/4057, 3.75%). We recruited a larger percentage of people who self-identified as gay men via intercept than social media (*P*<.001) and a larger percentage of people who self-identified as lesbian or gay women, gender minorities, and other sexual minorities via social media (*P*<.001). The majority of the sample reported having some college education (2049/3976, 51.53%), followed by a high school education or less (1040/3976, 26.63%), and a college degree or greater (887/3976, 22.31%). Participants who reported attending high school or less (*P*<.001) or some college (*P*=.005) were more likely to be recruited via social media than intercept, and those who reported having a college education or greater were more likely to be recruited via intercept (*P*<.001).

**Table 2:** Eligibility and survey completion by recruitment method. The denominator for each row percentage is from the row above. Comparisons between counts for *eligible* and *completed survey* rows are horizontal only.

Stage of completion	Intercept	Social media	*P* value
Completed screener, n	7375	6611	N/A^a^
Eligible, n (%)	4608 (62.48)	3357 (50.78)	<.001
Completed survey, n (%)	2348 (50.95)	1709 (50.90)	.97

^a^N/A: not applicable.

The sample was primarily white, non-Hispanic (1856/4057, 45.75%); followed by Hispanic (1211/4057, 29.85%), other or multiracial, non-Hispanic (587/4057, 14.47%); and black, non-Hispanic (403/4057, 9.93%). Black, non-Hispanic (*P*=.002) and Hispanic participants (*P*=.01) were more likely to be recruited via intercept, whereas white, non-Hispanic participants were more likely to be recruited via social media (*P*=.001).

On average, participants reported smoking cigarettes on 14 of the past 30 days (SD 11.73). Past 30-day smoking was higher among participants recruited via intercept (*P*=.02). About one-quarter of participants reported using alcohol on 3 to 5 of the past 30 days (904/3487, 25.92%), followed by 6 to 9 (849/3487, 24.35%), 10 to 19 (764/3487, 21.91%), 1 or 2 (671/3487, 19.24%), 20 to 29 (249/3487, 7.14%), and 30 of the past 30 days (50/3487, 1.43%). Participants who consumed alcohol on 1 or 2 (*P*<.001) or 3 to 5 of the past 30 days (*P*=.05) were more likely to be recruited via social media, whereas those who consumed alcohol on 10 to 19 or 20 to 29 of the past 30 days were more likely to be recruited via intercept (*P*<.001). The majority of participants reported past-year pride event attendance (2591/3092, 83.80%). A larger percentage of participants recruited via social media reported past-year pride event attendance (*P*<.001).

Social media use was high across the overall sample, with more than half of participants reporting Facebook use several times a day (2666/3943, 67.61%) and half of participants reporting Instagram use several times a day (1952/3942, 49.52%). Among those who reported using Facebook about once a day, a larger percentage were recruited via social media (*P*=.02). Among those who reported using Facebook less than once a day, a larger percentage were recruited via intercept (*P*=.006). Finally, among participants who reported using Instagram less than once a day, a larger percentage were recruited via social media (*P*=.02).

### Bivariate Analyses

Bivariate analyses (reported in text only) revealed that, as age increased, odds of recruitment via social media decreased (odds ratio, OR 0.73, 95% CI 0.71-0.76, *P*<.001). Lesbian or gay women (OR 1.80, 95% CI 1.53-2.12, *P*<.001), bisexual men and women (OR 1.43, 95% CI 1.22-1.69, *P*<.001), gender minorities (OR 2.08, 95% CI 1.65-2.62, *P*<.001), and other sexual minorities (OR 3.64, 95% CI 2.57-5.16, *P*<.001) were more likely than gay men to be recruited via social media. Participants with a high school education or less (OR 2.08, 95% CI 1.73-2.51, *P*<.001) and with some college education (OR 1.80, 95% CI 1.52-2.13, *P*<.001) were more likely than those with at least a college degree to be recruited via social media. Hispanic (OR 0.79, 95% CI 0.69-0.92, *P*=.002) and black, non-Hispanic participants (OR 0.66, 95% CI 0.53-0.83, *P*<.001) were less likely than white, non-Hispanic participants to be recruited via social media. Participants reporting past 30-day smoking (OR 0.84, 95% CI 0.74-0.95, *P*=.006) and drinking alcohol (OR 0.25, 95% CI 0.20-0.31, *P*<.001) were less likely to be recruited via social media. Participants who had attended a pride event in the past year were more likely to be recruited via social media (OR 1.48, 95% CI 1.21-1.81, *P*<.001), as were those who used Facebook at least once a day (OR 1.28, 95% CI 1.08-1.53, *P*=.005). In contrast, participants who reported using Instagram at least once a day were less likely to be recruited via social media (OR 0.85, 95% CI 0.74-0.97, *P*=.01).

**Table 3:** Sample characteristics.

Characteristic		Total sample (N=4057)	Social media (N=1709)	Intercept (N=2348)	*P* value
Age (years), mean (SD)		21.54 (1.87)	20.94 (1.94)	21.98 (1.69)	<.001
**LGBT^a^ identity, n (%)**					
	Gay men	1822 (44.91)	630 (36.86)	1191 (50.75)	<.001
	Lesbian or gay women	882 (21.74)	430 (25.16)	453 (19.30)	<.001
	Bisexual men and women	858 (21.14)	370 (21.65)	488 (20.79)	.51
	Gender minorities	342 (8.43)	179 (10.47)	163 (6.94)	<.001
	Other sexual minorities	152 (3.75)	100 (5.85)	52 (2.21)	<.001
**Education, n (%)**					
	High school or less	1040 (26.63)	489 (29.76)	551 (23.62)	<.001
	Some college	2049 (51.53)	889 (54.11)	1160 (49.72)	.005
	College plus	887 (22.31)	265 (16.13)	622 (26.67)	<.001
**Race or ethnicity, n (%)**					
	White, non-Hispanic	1856 (45.75)	832 (48.68)	1024 (43.61)	.001
	Black, non-Hispanic	403 (9.93)	141 (8.25)	262 (11.16)	.002
	Hispanic	1211 (29.85)	475 (27.79)	736 (31.35)	.01
	Other or multiracial, non-Hispanic	587 (14.47)	261 (15.27)	326 (13.88)	.23
Past 30-day cigarette use (N=1833), mean (SD)		13.54 (11.73)	12.73 (11.33)	14.07 (11.96)	.02
**Past 30-day alcohol use, n (%)**					
	1 or 2 days	671 (19.24)	343 (26.61)	328 (14.92)	<.001
	3-5 days	904 (25.92)	366 (28.39)	538 (24.48)	.05
	6-9 days	849 (24.35)	302 (23.43)	547 (24.88)	.33
	10-19 days	764 (21.91)	199 (15.44)	565 (25.71)	<.001
	20-29 days	249 (7.14)	64 (4.97)	185 (8.42)	<.001
	30 days	50 (1.43)	15 (1.16)	35 (1.59)	.28
Past-year pride event attendance, n (%)		2591 (83.80)	1113 (86.81)	1478 (81.66)	<.001
**Facebook use frequency, n (%)**					
	Several times a day	2666 (67.61)	1095 (67.97)	1571 (67.37)	.78
	About once a day	639 (16.20)	287 (17.82)	352 (15.09)	.02
	Less than once a day	638 (16.18)	229 (14.21)	409 (17.54)	.006
**Instagram use frequency, n (%)**					
	Several times a day	1952 (49.52)	776 (48.17)	1176 (50.45)	.16
	About once a day	563 (14.28)	215 (13.35)	348 (14.93)	.17
	Less than once a day	1427 (36.20)	620 (38.49)	807 (34.62)	.02

^a^LGBT: lesbian, gay, bisexual, and transgender.

**Table 4:** Multivariate logistic regressions of lesbian, gay, bisexual, and transgender (LGBT) young adults recruited via social media (vs intercept). Predictors include variables related to the recruitment methods in the bivariate analyses (*P*<.25). Variables for past 30-day cigarette and alcohol use are dichotomized to any past 30-day use vs no past 30-day use (reference category, REF). Social media use frequency variables are dichotomized to at least once a day vs less than once a day (REF). Analytic N=2945 (social media N=1183, intercept N=1762).

Variable		AOR^a^	95% CI	*P* value
Age		0.76	0.72-0.80	<.001
**LGBT^b^ identity**				
	Gay men	*REF^c^*	*REF*	*REF*
	Lesbian or gay women	1.88	1.54-2.29	<.001
	Bisexual men and women	1.46	1.17-1.82	.001
	Gender minorities	1.68	1.26-2.25	<.001
	Other sexual minorities	2.48	1.62-3.80	<.001
**Education**				
	High school or less	1.07	0.83-1.40	.60
	Some college	1.27	1.03-1.56	.03
	College plus	*REF*	*REF*	*REF*
**Race or ethnicity**				
	White, non-Hispanic	*REF*	*REF*	*REF*
	Black, non-Hispanic	0.76	0.58-1.01	.05
	Hispanic	0.73	0.61-0.89	.001
	Other or multiracial, non-Hispanic	0.70	0.54-0.90	.006
Past 30-day cigarette use		0.94	0.80-1.10	.42
Past 30-day alcohol use		0.33	0.24-0.44	<.001
Past-year pride event attendance		1.31	1.06-1.64	.02
Facebook use at least once a day		1.43	1.14-1.80	.002
Instagram use at least once a day		0.73	0.62-0.86	<.001

^a^AOR: adjusted odds ratio.

^b^LGBT: lesbian, gay, bisexual, and transgender.

^c^REF: Reference category.

### Logistic Regression Analyses

We present results from the final multivariate logistic regression in Table 4. As age increased, odds of recruitment via social media decreased (AOR 0.76, 95% CI 0.72-0.80, *P*<.001). Lesbian or gay women (AOR 1.88, 95% CI 1.54-2.29, *P*<.001), bisexual men and women (AOR 1.46, 95% CI 1.17-1.82, *P*=.001), gender minorities (AOR 1.68, 95% CI 1.26-2.25, *P*<.001), and other sexual minorities (AOR 2.48, 95% CI 1.62-3.80, *P*<.001) were more likely than gay men to be recruited via social media. Hispanic (AOR 0.73, 95% CI 0.61-0.89, *P*=.001); black, non-Hispanic (AOR 0.76, 95% CI 0.58-1.01, *P*=.05); and other or multiracial, non-Hispanic participants (AOR 0.70, 95% CI 0.54-0.90, *P*=.006) were less likely than white, non-Hispanic participants to be recruited via social media. Participants with some college education (AOR 1.27, 95% CI 1.03-1.56, *P*=.03) were more likely than those with at least a college degree to be recruited via social media.

Participants reporting past 30-day alcohol use were less likely to be recruited via social media (AOR 0.33, 95% CI 0.24-0.44, *P*<.001). Past 30-day smoking was not related to the likelihood of being recruited via social media vs intercept. Participants who reported past-year pride event attendance were more likely to be recruited via social media (AOR 1.31, 95% CI 1.06-1.64, *P*<.05) as were those who used Facebook at least once a day (AOR 1.43, 95% CI 1.14-1.80, *P*=.002). Participants who reported using Instagram at least once a day were less likely to be recruited via social media (AOR 0.73, 95% CI 0.62-0.86, *P*<.001).

## Discussion

### Principal Findings

Overall, our findings indicate that innovative recruitment methods that reached hard-to-reach populations in the virtual and physical spaces where they spend time were an effective means of recruiting LGBT young adults for FDA’s *This Free Life* campaign evaluation. Social media participants were younger and less educated (ie, more likely to report some college education than having a college degree or greater) compared with participants recruited via intercept. Social media participants were more likely to self-identify as lesbian or gay women, bisexual men or women, gender minorities, or other sexual minorities than as gay men compared with intercept participants. Social media participants were also more likely to be white, non-Hispanic than racial or ethnic minorities compared with intercept participants. Social media participants were more likely to have attended a pride event in the past year than were intercept participants. Finally, participants reporting past 30-day alcohol use were less likely to be recruited via social media than intercept.

Findings specific to age, LGBT identity, education, and alcohol consumption were not unexpected for several reasons. First, LGBT social venues and events tend to disproportionately cater to gay men in comparison with other LGBT subgroups (eg, lesbian or gay women). Many LGBT social venues are also restricted to those aged 21 years and older because they serve alcoholic beverages. Thus, attendance is not only limited to older individuals who are of legal drinking age but may also be limited to those with higher levels of education who are more likely to be able to afford drinking in bars and nightclubs. The highly specific targeting tools provided by social media platforms enabled us to recruit particularly hard-to-reach subgroups of LGBT young adults (eg, gender and other sexual minorities). In combination, our findings suggest that the unique features of the two recruitment methods complemented one another, allowing us to recruit a more balanced population of LGBT young adults. For example, the targeting tools available via social media advertising platforms allowed us to recruit a broader age range of LGBT young adults from hard-to-reach LGBT subgroups, whereas the tendency for LGBT social venues to be 21 years-and-over bars and nightclubs allowed us to recruit higher-risk LGBT young adults (ie, those who consume more alcohol and smoke more cigarettes).

One unexpected finding was that social media participants were more likely to be white, non-Hispanic than racial or ethnic minorities (ie, Hispanic, black, non-Hispanic) compared with intercept participants. It is unclear why this difference emerged given that white and non-white adults’ level of social media use is about equal [42]. White, non-Hispanic individuals have higher levels of at-home broadband internet access (82%) than black individuals (74%) [43], suggesting the possibility that a larger number of white, non-Hispanic participants recruited via social media ads completed surveys because they had a greater opportunity to click on social media ads and immediately proceed to completing the 30-min survey instrument while using social media at home. Because fewer racial or ethnic minorities have at-home broadband internet, they may have been more likely to see the social media ads while using smartphones on-the-go (ie, not at home), which may not have been an optimal time for completing the 30-min survey. It is unsurprising that this was not the case for intercept as eligible participants were emailed the survey link to complete at their convenience.

Although social media participants were more likely to use Facebook at least once a day, one counterintuitive finding was that social media participants were less likely to use Instagram at least once a day compared with intercept participants. It is important to note that, at the time of recruitment, Instagram advertising was newly available to researchers and has not yet been used extensively for participant recruitment. Although the reasons for this finding are unclear, it suggests that there may be important differences in recruitment methods that are most effective for recruiting Facebook and Instagram users. Furthermore, these differences may vary depending on frequency of use of each platform. We will explore this possibility in future research.

Beyond recruiting a diverse sample of LGBT young adults, both recruitment methods resulted in high eligibility rates among participants who completed the screening instrument. Eligibility was higher among intercept than social media participants, suggesting that intercept interviews are more likely to identify eligible members of the target population. Social media ads may reach a wider population beyond those who are LGBT, whereas more LGBT social venue attendees may be LGBT. Survey completion rates among eligible participants were similar between recruitment methods. This finding is promising for social media recruitment as a data collection method—previous research has shown that collecting data entirely via the Web leads to lower levels of participant accountability, which results from higher levels of psychological distance between researcher and participant in Web-based studies (compared with studies involving some level of face-to-face contact between researcher and participant) [44-46]. Rather than affecting completion rates, this psychological distance may instead have played out in terms of data quality. Data collected via social media were more vulnerable to low-quality and duplicate responses from participants trying to complete the survey more than once for additional incentives. In comparison, we needed to throw out only a nominal percentage of intercept surveys because of interviewer misrepresentation (ie, fake responses generated by interviewers).

Although intercept recruitment resulted in higher eligibility and had fewer data quality issues, from a practical perspective, social media recruitment was significantly more time efficient than intercept. From a proportion of recruitment costs perspective, social recruitment was also less expensive than intercept. We completed social media data collection in all markets in less than 2 weeks for the nominal cost of posting ads. In contrast, for manageability considerations, we launched intercept collection in markets on a rolling basis over the course of 7 weeks with two to five markets being launched in any given week. Due to wide variation by market in the volume of LGBT young adults present at local LGBT venues, intercept data collection lasted anywhere from 1 week to 9 weeks within each market. Slow or lengthy data collection in some markets reduced data collector morale and retention, requiring the expense of training additional local data collectors or temporarily relocating data collectors from other areas. From a cost perspective, intercept methods involve wages and other expenses associated with data collector time spent intercepting respondents and the expense of recruiting, training, and managing those data collectors. Furthermore, intercept respondents were provided with an additional US $10 cash incentive for taking the eligibility screener and a US $5 bonus for completing the main survey within 2 days of receiving the first invitation. Social media respondents were not offered additional incentives because they completed the screener after clicking on ads, and eligible participants proceeded directly to the main survey.

### Limitations

Although our research provides important insights about how samples can be recruited via innovative methods, we acknowledge several important limitations. First, samples that were recruited both via social media and intercept were convenience, nonprobability samples. Second, it is possible that participants were exposed to both recruitment methods more than once, which may have influenced their decision to participate in the study and was not measured in this study. Third, we were unable to conduct significance tests to compare costs between social media and intercept recruitment because cost data was available only as a proportion of total recruitment cost for each recruitment method. Fourth, the higher monetary incentive offered to participants recruited via intercept (ie, US $10 incentive for completing the screener and potential US $5 early survey completion bonus for main survey) was a confounding factor that may have influenced recruitment rates between methods. A final limitation was the difference in the method for approaching potential participants to complete the screening instrument via social media vs intercept. Intercept respondents were approached by data collectors to complete the screening instrument in person and may have experienced more perceived pressure to complete the screener than those recruited via social media who were shown ads and could choose whether or not to click on ads without being physically monitored by a third party.

### Comparison With Prior Work

Our research marks the recruitment of one of the largest samples of LGBT young adults to date. This research provides important contributions to the literature on using novel methods to recruit hard-to-reach populations; LGBT young adults in particular. Although previous studies have used social media to recruit members of the LGBT community, such as gay men [33,38-39], gay and bisexual male youth [30], and transgender women [47], our study demonstrates that social media can be used to recruit large numbers of particularly hard-to-reach and underrepresented LGBT subgroup members (eg, lesbian or gay women, bisexual men and women, gender and other sexual minorities).

In a similar vein, our research shows that large numbers of LGBT young adults in a number of different regions of the United States can be recruited in-person in social venues. These findings provide important contributions to the existing literature that has shown that these methods can be used to recruit LGBT young adults in bars and nightclubs in a single market [23] and young adults who are at a higher risk for smoking and alcohol use in a number of markets in the United States [24-28].

Our research also shows that a large and diverse sample of LGBT young adults can be recruited using Facebook and Instagram ads. To the best of our knowledge, all of the published literature demonstrating the effectiveness of using social media for participant recruitment has focused on using Facebook to recruit LGBT samples [33,38-39,47]. Instagram ads are somewhat newly available to researchers (since September 2015) [48] and show promise for recruiting hard-to-reach populations such as young adults, who have much higher representation on Instagram (59% of adults aged 18-29 years) than adults over the age of 30 years (33% of adults aged 30-49 years, 18% of adults aged 50-64 years, and 8% of adults aged 65+ years) [49]. Indeed, Instagram ads have rapidly evolved and now offer similar capabilities as Facebook ads because they use the same platform. These capabilities make Instagram ads a seamless tool to use alongside Facebook ads for recruitment.

Finally, this study shares explicit methodological details regarding our development of social media ads and strategy. This information may be particularly useful for researchers who seek to implement these tools in their own studies. Few published studies provide this level of detail on their social media recruitment methods [32].

### Conclusions

Novel methods that reach hard-to-reach populations such as LGBT young adults where they frequently spend time are effective participant recruitment strategies. We recruited LGBT young adults in LGBT social venues and via Facebook and Instagram ads. Using these methods in combination, we recruited a more diverse sample of LGBT young adults from a broader range of LGBT identities, race or ethnicities, ages, and education levels than we could have using either method in isolation. Importantly, social media ads provided enhanced access to particularly hard-to-reach subpopulations of LGBT young adults (ie, bisexual, gender, and sexual minorities) who were less easily accessed via intercept recruitment. Although social media data collection is a more efficient and inexpensive recruitment method, it is more subject to data quality issues than intercept data collection. Together, these methods enabled recruitment of one of the largest known LGBT young adult samples, suggesting their promise for recruiting hard-to-reach populations.

## Abbreviations

AOR: adjusted odds ratio
CTP: Center for Tobacco Products
DMA: designated market area
FDA: US Food and Drug Administration
GPS: global positioning system
IP: internet protocol
IRR: incidence rate ratio
LGBT: lesbian, gay, bisexual, and transgender
OR: odds ratio
REF: reference category
